# Penile Osteosarcoma: A Rare Extraosseous Site

**DOI:** 10.7759/cureus.56050

**Published:** 2024-03-12

**Authors:** Kashish Khanna, Kyle Michelson, Daniel P Pierce, Trushar Patel

**Affiliations:** 1 Urology, University of South Florida Morsani College of Medicine, Tampa, USA

**Keywords:** extra-skeletal osteosarcoma, high-grade sarcoma, bilateral inguinal lymph node dissection, penile pain, partial penectomy, penile cancer, penile mass

## Abstract

Primary penile extraosseous osteosarcoma (EOS) ranks the most uncommon amongst the differential penile masses, with only nine cases reported so far. In this report, we share the management of a 67-year-old Hispanic male who presented with a painful mass over his distal penile shaft and glans for the last two months. After initial imaging and complete blood investigations, he underwent partial penectomy. Histology revealed high-grade sarcoma, with osteoid production, favoring high-grade extra-skeletal osteosarcoma, with tumor necrosis involving approximately 5% of the tumor volume. The patient had bilateral palpable inguinal lymphadenopathy, which was seen even on a pre-op CT scan. The patient thus underwent bilateral robotic superficial and deep inguinal standard template lymph node dissection three weeks after his partial penectomy. His pathology was negative for malignancy in all examined lymph nodes. At his last follow-up, five months post his primary surgery, he had been doing well without concerns for recurrence.

## Introduction

Extraosseous osteosarcoma (EOS) may arise from various sites such as the retroperitoneum and the muscles of thighs or limb girdles. Rarely, it has also been described in the lung, prostate, scalp, mammary gland, spermatic cord, pelvis, and orbit. Among these sites, primary penile EOS ranks as the most uncommon, with only nine cases reported in the literature [1-9], and the youngest reported patient is a 19-year-old boy [9]. EOS expands an already rare differential for masses of the genitalia. The main differential for its presentation is the dreaded penile cancer. However, EOS, if diagnosed in time, can be managed successfully by surgical resection, which may entail complete or partial penectomy [1-9]. The five-year survival of these patients varies between 25 and 37% [8], mostly because of the metastasis rate of this tumor. Owing to the rarity of an EOS of the penis, there has been no standardized management protocol following surgery. Some reports of postoperative chemotherapy have been shown to be beneficial [7]. Below, we present a patient with a penile mass where histology confirmed the rare diagnosis of penile osteosarcoma. Our management differs from that described in the literature as we also performed a robotic bilateral inguinal lymph node dissection to improve survival, decrease morbidity, and decrease the likelihood of metastasis after partial penectomy in our patient.

## Case presentation

**Figure 1:**
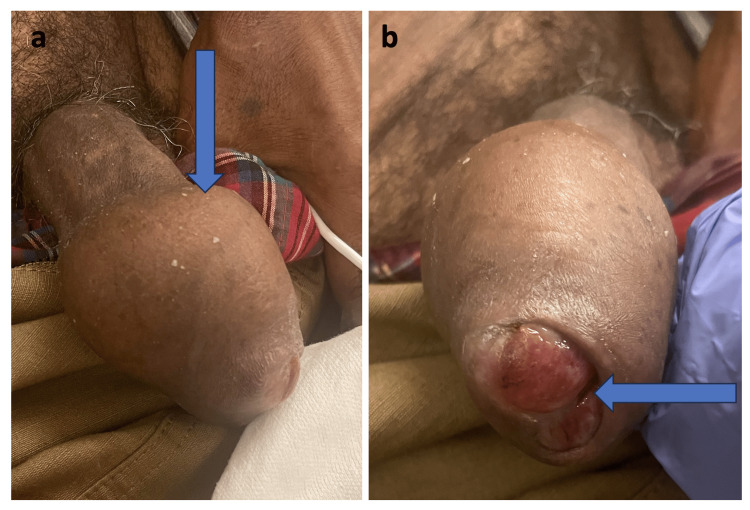
Clinical picture a: A 7 cm growth over the distal penile shaft with irregular margins, firm consistency, and nontender, sparing greater than a 2 cm region at the base of the penis. b: End on appearance showing obliteration of urethral meatus.

A Hispanic male in his late 60s presented to the emergency department with a painful mass involving his distal penile shaft and glans two months before presentation, which had increased significantly in size. He denied a history of any similar swelling or lesions in the past, recent penile trauma, instrumentation, radiation, recurrent urinary tract infections, sexually transmitted diseases, penile discharge, history of men having sex with men (MSM), or unprotected intercourse. He denied any issues with urination, bone pains, jaundice, respiratory discomfort, or dizziness. He reported no history of cancer. He is a chronic smoker and a type 2 diabetic on oral hypoglycemic agents. A genitourinary exam revealed a large mass on the distal end of his uncircumcised penis, approximately 7 cm in size. The urethral meatus could not be identified (Figure 1). There were a few palpable sub-centimetric left inguinal lymph nodes, which were nontender, firm, and mobile. Both testes were descended in the scrotum and were symmetrical without any appreciable induration.

**Figure 2:**
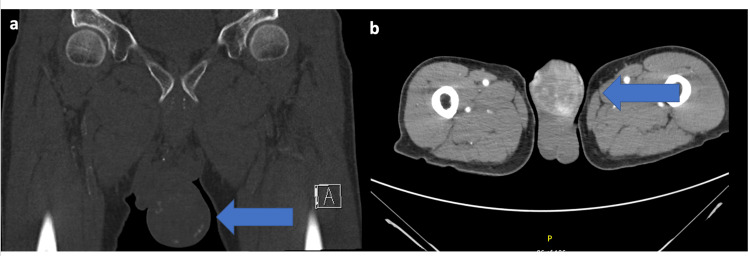
Computed tomography scan showing the heterogenous penile mass with areas of calcifications a. Coronal section; b. Axial section.

His routine blood work was unremarkable. The labs exhibited no leukocytosis, hemoglobin within normal range, normal creatinine, and unremarkable liver function tests. His urine analysis was negative for infection and inflammation. A computed tomography (CT) scan of the pelvis without intravenous contrast was performed and showed an abnormal penile mass at the distal aspect of the shaft with variable densities and areas of calcification, measuring approximately 7 cm (Figure 2). This was concerning for malignancy especially given the confirmation of bilateral inguinal lymphadenopathy. His chest CT scan was normal, and no other significant findings were noted on the abdominal/pelvic CT scan. These findings were consistent with the physical exam findings.

The working diagnosis was primary malignancy of the penile shaft and glans of likely squamous or human papillomavirus (HPV) origin in the setting of possibly locally advanced disease with bilateral palpable inguinal lymphadenopathy and no evidence of distant metastasis on imaging. This was supported by the patient’s rapid increase in the size of his penile mass in a short period, palpable inguinal nodes, no previous history of malignancy, presenting signs and symptoms, and lab work, suggesting no infectious etiology.

Because of the high suspicion of malignancy, rapidity of growth, and obstruction of the urethral meatus, the patient agreed to urgent surgical intervention within the same admission. We explained to the patient the role of upfront inguinal lymph node dissection given the concern for lymphadenopathy on exam and CT imaging. Moreover, it was emphasized that 25% of patients with negative exam/imaging could still harbor disease, especially in high-risk groups, including those with clinical T3 disease, lymphovascular invasion, and high-grade tumors. The patient was concerned about associated morbidity with open inguinal lymphadenectomy, but he was interested in an attempted robotic-assisted inguinal lymph node dissection; however, this could not be offered at the initial presentation. Therefore, two days after a detailed discussion with the patient regarding the risks and benefits of surgery, he was taken for partial penectomy with the decision to delay regional lymphadenectomy. Intraoperative findings were notable for a large 7 cm tumor involving the distal shaft and glans with overall clean margins free of tumor and greater than a 2 cm penile stump, which was deemed to be more than adequate for functional use. The urethra overall was widely approximated and permitted easy passage of a Foley catheter. Postoperatively, the patient did well, the dressing was removed on post-op day 1, and he was discharged with a Foley catheter, which was removed after one week in the clinic. His partial penectomy wound healed appropriately.

**Figure 3:**
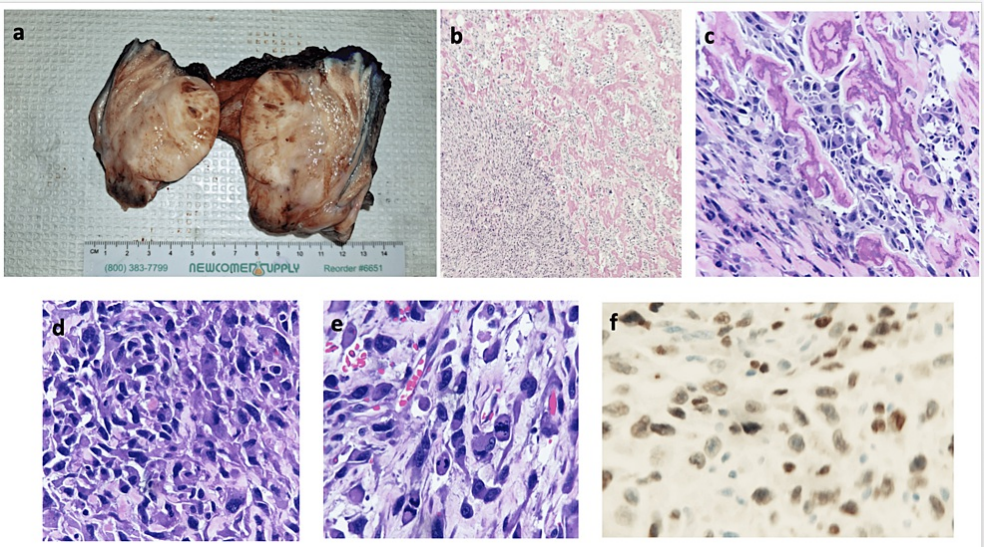
Histological findings in extraosseous penile osteosarcoma a. Gross picture, b. low magnification, c. medium magnification with osteoid production, d. high magnification in more cellular areas, e. many mitoses, f. SATB2-positive nuclear stain.

The pathological findings of the specimen were high-grade sarcoma, with osteoid production, favoring high-grade extraskeletal osteosarcoma, with tumor necrosis involving approximately 5% of the tumor volume. Immunostains demonstrated that the tumor cells were positive for vimentin, SATB2, and variably positive for SMA and podoplanin; tumor cells were negative for pankeratin, CAM5.2, EMA, desmin, S100, Sox10, CD34, LCA, CD68, and β-catenin (negative for nuclear stain). The greatest tumor dimension was 5.5 cm, the mitotic rate was approximately 10/mm2, and the histologic grade was 3 (FNCLCC). All margins were free of the tumor; the proximal skin resection margin was 0.5 cm from the tumor; the proximal soft tissue resection margin was 0.7 cm from the tumor and 1.6 cm to the urethral resection margin, all of which were considered adequate for negative margin status. Hence, the soft tissue sarcoma pathologic staging classification was Stage IIIA pT2, pNx, pMx, and G3. The mass grossly appeared to replace approximately 95% of the right corpus cavernosa and 85% of the left corpus cavernosum. The mass exhibited pushing borders, which approached within 0.2 cm of the grossly uninvolved corpus spongiosum muscle. The mass entirely abutted the Dartos fascia with no gross infiltration. Moreover, the mass extended to replace approximately 70% of the total glans (Figure 3).

During the follow-up visit, we again discussed the need for a follow-up inguinal lymphadenectomy. Because of the rarity of primary EOS of the penis, the role of lymph node dissection is not well delineated as is the case with penile cancer. Literature shows that early inguinal lymphadenectomy in clinically node-negative patients is superior for long-term patient survival compared to therapeutic lymphadenectomy when regional nodal recurrence occurs [10]. The decision to proceed with inguinal lymph node dissection in the setting of EOS of the penis was made after consultation with our tumor board. Additionally, it was believed that the lymph node dissection could be performed robotically, which would reduce the overall morbidity of the procedure. The patient agreed to proceed with bilateral robotic superficial and deep inguinal standard template lymph node dissection three weeks after his partial penectomy. The patient had an uneventful immediate postoperative course and was discharged home the following day. His bilateral inguinal drains were removed after two weeks, and his pathology was negative for malignancy in all examined lymph nodes. The patient has been followed regularly in the clinic, and at his last follow-up, five months post his index surgery, he has been doing well without concerns for recurrence.

## Discussion

Penile lesions incorporate a broad spectrum of conditions, of which penile cancer is the most dreaded. The above patient presented primarily because of pain in his phallus, which had been present for about two months. Primary osteosarcoma of the penis, a form of EOS, is an extremely rare cancer with only nine case reports in literature so far [1-9], accounting for only 1% of soft tissue sarcomas. Its risk factors are not well established. This most commonly affects men aged 45-65 [7,11]; however, it has recently been reported in as young as a 19-year-old boy [9]. Some suspected risk factors for EOS include trauma, radiation, and dermatomyositis [12]. The above-reported patient was Hispanic, uncircumcised, and a chronic smoker; however, he had no history of radiation or trauma. Swelling and pain in the penile lesion are common presenting complaints because of the rapid growth of these tumors [8]. The diagnostic criteria of EOS include the following: 1) it is found in soft tissue and is not attached to bone or periosteum; 2) it has a uniform sarcomatous pattern (to exclude the possibility of a mixed malignant mesenchymal tumor); and 3) producing osteoid or cartilaginoid matrix [13]. EOS are mostly high-grade with malignant osteoid and have cartilaginous elements [6,7]. Athanazio et al. reported atypical mononuclear cells with diffusely positive special adenine- and thymine (AT)-rich sequence-binding protein 2 (SATB2), a marker of osteoblastic differentiation, cluster of differentiation 99 (CD99), and vimentin, and they concluded that SATB2 staining may be useful for identifying EOS [9]. In the reported case, the SATB2 nuclear staining is positive; however, unfortunately, alkaline phosphatase staining was not specifically performed on this specimen. Sections demonstrated a hypercellular malignant neoplastic proliferation with pleomorphic cells including spindled, epithelioid, rhabdoid, signet ringlike, and multinucleated tumor cells. Osteoid production with mineral deposition/calcium deposition is identified in the tumor stroma. Osteoclast-like multinucleated cells were also present, favoring osteosarcoma primary rather than penile cancer with osseous elements.

Treatment of these penile EOS tumors is initially surgical. Of the reported cases in the literature, three underwent partial penectomy without lymph node dissection, one underwent excision of the mass, two underwent complete penectomy, and one with a lesion at the glans was treated by partial glansectomy with glansplasty [1-9]. One received post-op chemotherapy, cisplatin based [7]. EOS are mostly radioresistant and do not respond well to radiotherapy. So far, only one patient has been reported to be alive at more than three years' follow-up. More than 75% of these patients have local or distant metastasis, resulting in death [7,8]. In the reported patient, a robotic bilateral superficial and deep inguinal lymphadenectomy was performed three weeks after the completion of the partial penectomy. This was based on treatment principles of penile cancer considering the high rate of metastasis within five years reported in the literature, including lung metastasis [9]. At the time of lymphadenectomy, given that the patient had multiple palpable nodules, it was decided that both a superficial and deep lymph node dissection would be performed as both compartments were easily accessible given the robotic approach, and there was a high index of suspicion for clinically positive disease. Frozen sections, therefore, were not sent for analysis, and all packets were carefully removed macroscopically, all of which proved to be negative for malignancy. Wu et al. reported recurrence, transfer, and five-year survival rates as 45%, 65%, and 25%-37%, respectively [8]. It was highly debated during the tumor board presentation if this patient should be managed based on soft tissue sarcoma guidelines, which would favor chemotherapy versus lymphadenectomy, favoring the management of primary penile cancer. Given this patient's limited medical resources, limited ability to follow-up, and absence of distant disease, he was preferably managed surgically. Additionally, given the patient's presumed high local disease burden, the role of sentinel lymph node biopsy was not explored as even if his primary node would have proven to be negative, the clinical suspicion was high enough that a complete dissection would have been performed and, thus, management would have remained unchanged. The above-reported patient is on regular follow-up and has been doing well at five months postoperatively; however, a longer follow-up will be required to look at the recurrence and five-year survival.

## Conclusions

The penis is an extremely rare site for extraosseous osteosarcoma, and most patients present with pain and swelling. Penile osteosarcomas are rapidly growing, high-grade tumors with no standard treatment protocol; however, in the absence of visceral or distant concern for metastasis, we recommend that treatment should be based on principles of penile cancer given the low rates of disease-free metastasis and five-year overall survival in these patients. Robotic inguinal lymphadenectomy in patients with palpable inguinal lymph nodes should be explored as an option to increase the five-year survival of these patients while lowering overall treatment morbidity.
